# Vial-splitting and Repackaging into Aliquot-specific Syringes: A Cost-effective and Waste-decreasing Strategy for Sugammadex

**DOI:** 10.1097/pq9.0000000000000646

**Published:** 2023-04-10

**Authors:** Sebastian Amaya, Sidhant Kalsotra, Nguyen K. Tram, Joseph D. Tobias, Vanessa A. Olbrecht

**Affiliations:** From the * Department of Anesthesiology and Pain Medicine, Nationwide Children’s Hospital, Columbus, Ohio; † Department of Anesthesiology & Pain Medicine, Ohio State University Wexner Medical Center, Columbus, Ohio.

## Abstract

Sugammadex is a medication that may have cost considerations with the potential for waste of unused product in pediatric patients due to the vial size and its single-use limitation. Therefore, exploring the potential of vial-splitting for perioperative use may be beneficial.

**Methods:**

The study was a retrospective, quality improvement study using the electronic medical record to identify every sugammadex administration over the last five years in a tertiary care pediatric institution. We divided patients into groups depending on the dose of sugammadex administered. The cost of sugammadex was calculated under three scenarios: (1) only 200-mg vials available; (2) 100-mg aliquots available; and (3) 50-mg aliquots. We then calculated the total money spent per patient in the 3 scenarios.

**Results:**

31,063 patients received sugammadex over the study period, of whom 23.6% received 151–200 mg. The greatest percentage of patients received ≤50 mg (32.9%). The average cost per patient was $113.58, $81.61, and $68.83 if 200 mg, 100 mg, and 50 mg doses were available, respectively. Over the last 5 years, $1,390,110.13 could have been saved by having 50 and 100 mg aliquots available.

**Conclusions:**

Pediatric patients generally receive lower doses of sugammadex due to weight-based dosing, leading to increased waste and cost when using only 200-mg vials. Vial-splitting into smaller aliquots can significantly cut costs for healthcare centers and patients while decreasing waste.

## INTRODUCTION

In general, pediatric patients tend to require less medication when compared with adults. This fact can lead to significant medication waste and increased costs for patients and health institutions, especially with medications that come in a single-use presentation, such as sugammadex. Although prices vary depending on purchasing agreements and other institutional and regional factors, the cost of a 200-mg single-use vial of sugammadex is ≥$90 per vial. Dosing based on manufacturer recommendations generally varies from 2 to 4 mg/kg depending on the depth of residual neuromuscular blockade needing reversal.^1^ With this dosing regimen, adult patients normally receive an entire vial; however, pediatric patients often receive less than an entire vial, leading to unnecessary waste of single-dose vials, thereby increasing institutional and patient costs. This possibility leads to the hypothesis that vial-splitting sugammadex into smaller, aliquot-specific syringes can generate significant savings. Various studies for other medications have shown a significant benefit with vial-splitting and drug repackaging, with decreased medication costs and less waste.^2,3^ In these cases, the pharmacy performs vial splitting and repackaging under a sterile hood to ensure patient safety. It is important to note that vials should not be empirically split inside the operating room, as this can increase the risk of contamination between patients and violates the current standard of care.

Exploring cost-efficiency strategies to offer the best care to our patients while optimizing resource use and cost is vital. We hypothesized that dividing standard 200 mg vials of sugammadex into smaller aliquots (50 mg and 100 mg) would yield significant cost savings at both the institution and patient levels. The current study retrospectively explores this possibility by examining dosing schemes at our institution.

## METHODS

This retrospective quality improvement study used the electronic medical record (Epic Systems, Verona, Wis.) to identify every sugammadex administration from November 2017 to November 2022. As a quality improvement project, the institutional review board waived the need for institutional review board approval. First, we stratified patients into 5 groups based on the dose of sugammadex received: ≤ 50, 51–100, 101–150, 151–200, and >200 mg. We then calculated the number of 200 mg vials, 100 mg aliquots, and 50 mg aliquots needed for each patient based on the dose. Next, we calculated the average amount of money spent per patient under 3 scenarios: (1) only 200 mg vials available; (2) 100 mg aliquots also available; and (3) 50 mg aliquots also available. We assumed that the cost of each 200-mg vial, 100-mg aliquot, and 50-mg aliquot of sugammadex was $106, $53, and $26.50, respectively, considering that a vial of sugammadex costs the pharmacy $106 at our institution. Subsequently, we multiplied the average amount of money spent per patient by the total number of patients in the dataset to produce the total amount spent in each scenario. Finally, we calculated the time required to redistribute contents within vials into syringes. We extrapolated these data to our study to calculate the full-time equivalent (FTE) staff necessary to split vials consistent with our usage over the 5-year study period.

## RESULTS

**Fig. 1.**
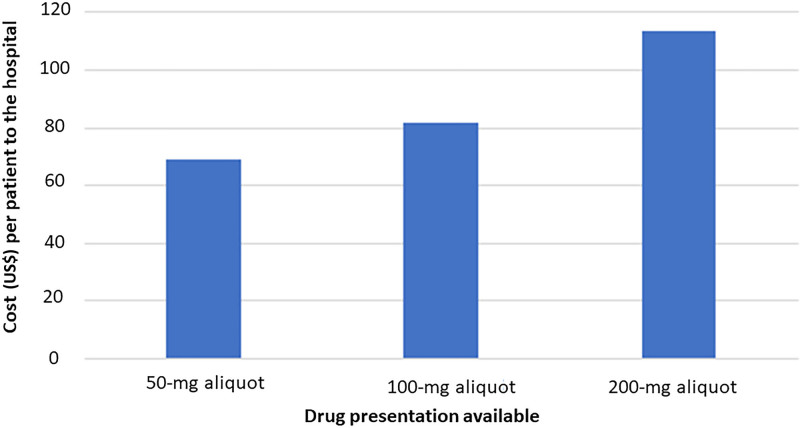
Average dollar cost per patient depending on drug presentation available.

**Fig. 2.**
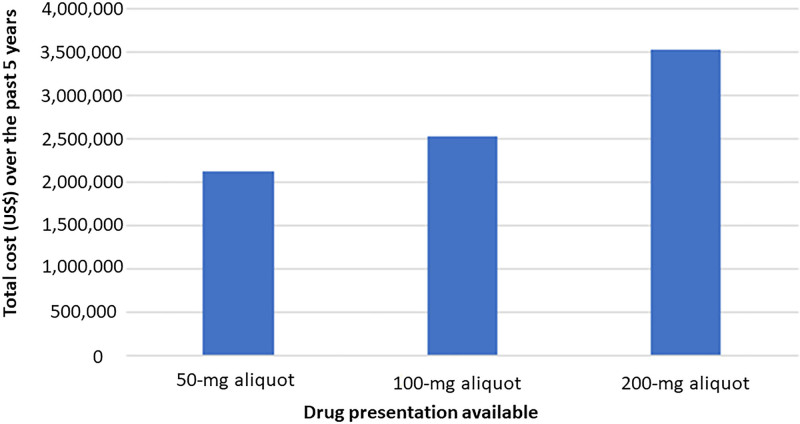
Total dollar cost over the past 5 years, depending on drug presentation available.

From November 2017 to November 2022, 31,063 patients received sugammadex in the operating room. Only 23.6% of cases received 151–200 mg, with the greatest number of patients receiving ≤50 mg (32.9%). The average cost per patient was $113.58, $81.61, and $68.83 if 200 mg, 100 mg, and 50 mg doses were available, respectively. The average cost when only a 200 mg vial is available is $113.58, considering that patients had to use the 200 mg vial regardless of whether or not they needed the whole vial, generating an automatic $106 cost (the cost per vial at our institution). However, some patients who required higher doses had to use 2 vials, thus increasing the average to $113.58 (and so on for the rest of the values). Over the last 5 years, $1,390,110.13 could have been saved simply by having 50 and 100 mg aliquots available (Table 1). Figures 1 and 2 show the changes in total and average costs per patient with available aliquots.

**Table 1.** Use and Cost Analysis of Sugammadex over 5 Years (n = 31.063)

**Amount of Sugammadex Used Per Case**	**Total Cases**	
≤50 mg	10,212 (32.9%)	
51–100 mg	7424 (23.9%)	
101–150 mg	4124 (13.3%)	
151–200 mg	7333 (23.6%)	
>200 mg	1970 (6.3%)	
**Doses Available**	**Average Cost (Dollars) to the Hospital per Patient**	**Total Cost over the Past 5 Years**
200-mg vial	$113.58	$3,528,108.76
100-mg aliquot	$81.61	$2,535,049.58
50-mg aliquot	$68.83	$2,137,998.63

With respect to the time taken to split vials at our institution, it takes approximately 2 minutes to separate one 20-mL vial of saline solution into two 10-mL syringes (including time taken for hand-washing and other sanitation techniques). Similarly, separating a 20 mL vial into one 10-mL and two 5-mL syringes, or a 20-mL vial into four 5-mL syringes also takes approximately 2 minutes. Extrapolating these data to our study allows us to estimate the time and cost of vial splitting. Considering that, on average, 6657 vials of sugammadex were used yearly during the study period (128 vials per week), and that it takes 2 minutes to repackage a vial, a total of 221.9 hours (4.27 hours weekly) is required to repackage the number of vials used, thus equaling approximately 0.1 (10.7%) FTE.

## DISCUSSION

Sugammadex has become widely used to reverse amino-steroid neuromuscular blocking agents such as rocuronium. Its mechanism of action and efficacy in various clinical scenarios have created a great demand for its use despite its cost, which is often more than $100 per vial. Sugammadex is currently prepackaged from the manufacturer as either 200 mg or 500 mg in a single-use vial. The recommended dose for reversal of neuromuscular blockade is 2−4 mg/kg depending on the depth of residual neuromuscular blockade as assessed using a train-of-four (TOF) monitor.^4^ As these vials are single-use, medication waste can occur, particularly in pediatric patients. The Centers for Disease Control recommends that vials that carry a single-use label, such as sugammadex, should only be used on a single patient during a single case, and thus do not recommend the use of the same vial for multiple patients, thus precluding splitting of the vial in the operating room by the anesthesia provider.^5^ Previous reports demonstrate the potential for infectious disease outbreaks stemming from healthcare professionals using a single-use vial on multiple patients.^6–8^ However, given dosing requirements in pediatric patients, if appropriate standards and sterile technique are followed, we believe that vial-splitting of sugammadex is a viable strategy to decrease waste and increase cost-efficiency, particularly in pediatric patients while abiding with current guidelines to avoid contamination and infection.

Due to their smaller size, pediatric patients often require smaller doses than adult patients. Our findings confirmed that the greatest number of patients received ≤50 mg (32.9%) during the study period (Table 1). Unfortunately, this fact increases costs and drug wastage, especially when using medications packaged in single-use vials such as sugammadex.

As the standard dose of sugammadex ranges between 2 and 4 mg/kg, most pediatric patients will not generally require the entire 200 mg vial leading to unnecessary waste and increased costs. We noted that the average cost of sugammadex per patient was $113.58, $81.61, and $68.83 if 200 mg, 100 mg, and 50 mg aliquots were available, respectively. When evaluated over the 5-year study period, using only the standard 200 mg vials of sugammadex was the most expensive, whereas having 50 and 100 mg aliquots available would generate significant savings (Fig. 2).

Consistent with our findings, other studies in various clinical contexts provide data supporting vial-splitting’s economic benefits. Gilbar et al distributed a survey to the members of the International Society of Oncology Pharmacy Practitioners and the British Oncology Pharmacy Association.^2^ The survey inquired what vial-sharing strategies were used, what means were employed to extend stability, how prepared products were reused, and what cost savings were achieved with this practice. The investigators reported significant drug wastage and the potential to decrease cost using vial sharing/splitting. However, they cautioned that it is important to have sufficient infrastructure and financial support to ensure proper protocols and processes for repackaging.^2^ Liu et al demonstrated decreased drug wastage of chemotherapeutic agents with intelligent dispensing robots and a vial-sharing strategy.^3^ Sodre et al reported that when compared with standard single vial use, vial sharing for the intravitreal administration of bevacizumab resulted in a decrease in public health costs and increased the availability of the drug to public health care patients.^9^

One important question to consider is the cost-effectiveness of repackaging medications into separate vials. Our calculations do not consider personnel costs and potential waste from expiring aliquots, as these aliquots generally have a shorter shelf-life than single-use vials. Kelm et al conducted a study comparing cost savings involving the repackaging of medications in various locations, including at the level of the drug manufacturer, third party companies, and in-house pharmacies. They found that the cost of repackaging was approximately the same no matter where it was done and was unlikely to significantly impact the pharmaceutical budget for a major health center.^10^ Heaton and colleagues found only a $0.05 increase per dose when repackaging drugs into units of use or medications per dose.^11^ This $0.05 increase would be significantly outweighed when compared with the amount of drug lost and associated cost when not using the entirety of a 200 mg vial of sugammadex. These results are consistent with our study findings, as we noted only a 0.1 FTE necessary to split sugammadex vials. Even if this process required a larger percentage of an FTE, the cost savings demonstrated per year by vial-splitting of sugammadex appears to justify the additional personnel cost and warrants further investigation into how this process can be implemented for this medication. In addition, it also highlights the need to expand the exploration of this practice for other costly medications that also carry significant potential for waste.

Although vial splitting sounds like an easy solution to improve costs and drug wastage, instituting this practice within a hospital may be challenging due to workforce and operational limitations. Our institution experiences a 20%–25% annual turnover rate for pharmacy technicians, and many institutions additionally face a 10%–20% vacancy rate for these positions. Ensuring limited vacancies and additional workforce would be required to initiate this process in day-to-day pharmacy operations. Current drug shortages have added to the challenge. Like many other hospitals, our pharmacy must manually compound syringes for other medications not previously required due to current drug shortages. As long as these shortages in the workforce and medications continue, significant challenges exist in dedicating time to splitting sugammadex vials due to these limitations.

It is also important to highlight some additional limitations of this study. First, as this was a retrospective chart review, we could not determine the cost to patients due to the complexity of insurance billing and the impact on out-of-pocket costs. We should also note that many anesthesia charges are bundled; thus, the costs of individual drugs are rarely included in pricing and transferred to the patient. Likewise, it is also important to consider the cost of the pharmacy personnel needed to split vials into smaller aliquots in addition to the challenge of availability of pharmacy staff. However, previous studies have shown only a minimal increase in pricing due to repackaging that does not outweigh the cost reduction generated from having smaller aliquots of medications available.^11^ In the current state of healthcare, not all institutions will have sufficient infrastructure or personnel readily available to split and repackage single-use vials. Additionally, we did not account for waste generated from aliquot syringes which is an important consideration given that pharmacy-made syringes generally have a shorter shelf-life than medication vials and can lead to unnecessary waste if not used within a certain time frame.

## CONCLUSIONS

Our study shows that by vial-splitting and decreasing the size of aliquots of sugammadex available for pediatric patients, there is the potential for a significant decrease in medication waste and notable cost savings. Strategies must be considered to mitigate costs associated with any medication, particularly in children who receive smaller doses. Our findings support that vial splitting may effectively decrease waste and cost associated with this medication.

## DISCLOSURE

The authors have no financial interest to declare in relation to the content of this article.
